# Left ventricular outflow tract obstruction after repair of atrioventricular septal defect

**DOI:** 10.1016/j.xjon.2023.06.009

**Published:** 2023-07-01

**Authors:** Edward Buratto, Igor E. Konstantinov

**Affiliations:** a Department of Cardiac Surgery, Royal Children's Hospital, Melbourne, Victoria, Australia; b Department of Paediatrics, University of Melbourne, Melbourne, Victoria, Australia; c Heart Research Group, Murdoch Children's Research Institute, Melbourne, Victoria, Australia; d Melbourne Centre for Cardiovascular Genomics and Regenerative Medicine, Melbourne, Victoria, Australia

To the Editor:

**Figure:**
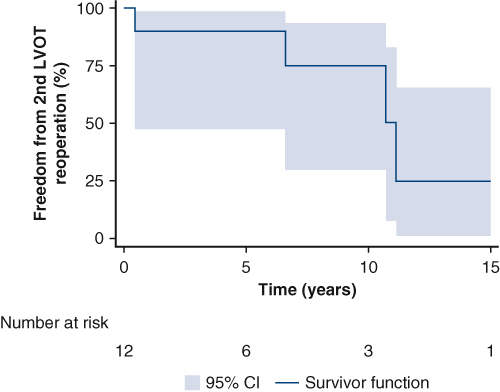

The authors reported no conflicts of interest.

The *Journal* policy requires editors and reviewers to disclose conflicts of interest and to decline handling or reviewing manuscripts for which they may have a conflict of interest. The editors and reviewers of this article have no conflicts of interest.

We read with interest the article by Chandiramani and colleagues^1^ on left ventricular outflow tract obstruction (LVOTO) after repair of complete atrioventricular septal defect (cAVSD). They observed that the subaortic area is intrinsically narrow in patients with cAVSD, and that the narrowing becomes more severe after complete repair, both with 2-patch and modified single patch (MSP) techniques. They noted that MSP technique was associated with a greater decrease in the subaortic diameter compared with the 2-patch technique. Despite this morphologic observation, the rates of reoperation were low in both groups and not statistically different. This mirrors the observation from data in Australia, where in a propensity score matched cohort there was no difference between 2-patch and MSP techniques in terms of reoperation.^2^ Possibly, the rarity of the reoperation for LVOTO means that both studies are underpowered to detect a difference between the groups. Furthermore, both studies lack data on LVOT gradients, and it is possible that the smaller LVOT observed in patients with MSP results in higher gradients and potentially greater LV afterload, with potentially deleterious effects on the LV diastolic function and left atrioventricular valve function. Thus, the narrowed subaortic region may not be benign, despite the lack of difference in reoperation rates between the groups.

This additional data may help refine our application of the 2 techniques. It has already been demonstrated that in the setting of a deep VSD that the MSP results in worse left atrioventricular valve function.^3^ An additional reason to choose 2-patch technique appears to be the presence of a narrower subaortic area pre-operatively.

We routinely adopt a 2-patch technique for repair of cAVSD for all but the shallowest VSDs and have observed a low rate of reoperation for LVOTO.^4^^,^^5^ When reoperation is needed, it is somewhat challenging, often requiring both transaortic and transatrial approaches, and has a high risk of requiring further interventions on the LVOT.^5^ Thus, the initial occurrence of LVOTO should be avoided whenever possible, and caution should be used when considering the MSP in patients with a narrow LVOT on preoperative imaging.
